# Scapular Alignment Restoration After Posterior Fixation of Displaced Scapular Fractures

**DOI:** 10.3390/medicina62071322

**Published:** 2026-07-09

**Authors:** Myung-Sub Lee, Doo-Hyung Lee, Jaeheon Lee, Won-Tae Cho, Seungyeob Sakong, Wan-Sun Choi

**Affiliations:** 1 Department of Orthopaedic Surgery, School of Medicine, Ajou University, Suwon 16499, Republic of Korea; mslee0618@naver.com (M.-S.L.); doolungee@gmail.com (D.-H.L.); ccarius85@gmail.com (W.-T.C.); sgsy4040@gmail.com (S.S.); 2 Department of Orthopaedic Surgery, Graduate School, Ajou University, Suwon 16499, Republic of Korea; leejaeheon.kr@gmail.com

**Keywords:** scapular fracture, internal fixation, posterior approach, lateral border offset, angular deformity

## Abstract

*Background and Objectives*: The surgical management of displaced scapular fractures remains controversial, particularly regarding the importance of restoring scapular alignment. This study evaluated radiologic restoration and clinical outcomes following posterior fixation of displaced scapular fractures, with a particular focus on correction of lateral border offset (LBO) and angular deformity. *Materials and Methods*: This retrospective case series included 20 patients who underwent posterior open reduction and internal fixation for displaced scapular fractures between 2017 and 2024 with a minimum follow-up of 12 months. Surgical indications included lateral border offset (LBO) > 20 mm, angular deformity > 30°, or displaced intra-articular fractures with step-off > 3 mm. Radiologic parameters including LBO, angular deformity, and intra-articular step-off were measured using computed tomography before and after surgery. Clinical outcomes were evaluated using shoulder range of motion (ROM), Disabilities of the Arm, Shoulder and Hand (DASH) score, and modified American Shoulder and Elbow Surgeons (ASES) score. *Results*: Significant postoperative improvement was observed in all radiologic parameters. Mean LBO improved from 18.9 ± 10.7 mm (range, 0–45.5 mm) to 3.1 ± 7.1 mm (range, 0–29.9 mm), angular deformity improved from 28.7° ± 11.3° (range, 10.2–48.1°) to 0.9° ± 3.9° (range, 0–17.8°), and intra-articular step-off improved from 6.4 ± 2.0 mm (range, 3.7–9.7 mm) to 1.8 ± 0.5 mm (range, 1–2.5 mm). At final follow-up, mean forward flexion was 126° ± 34.4° (range, 10–170°) and external rotation was 62.3° ± 20.8° (range, 0–90°). Mean DASH and modified ASES scores were 27.3 ± 17.3 (range, 8.3–74.2) and 71.6 ± 15.0 (range, 25.8–89), respectively. Glenoid involvement was not associated with inferior clinical outcomes, whereas associated ipsilateral upper extremity injuries tended to be related to poorer functional results. *Conclusions*: Posterior fixation effectively restored scapular alignment and articular congruity in displaced scapular fractures. Restoration of LBO and correction of angular deformity may represent important surgical objectives for correction of glenoid medialization and restoration of normal scapular alignment.

## 1. Introduction

Scapular fractures are uncommon, accounting for approximately 1% of all fractures and 3–5% of shoulder girdle fractures [1,2,3]. Because the scapula serves as a structural link between the axial skeleton and the upper extremity, disruption of its anatomy may affect shoulder mechanics and function. These injuries are frequently associated with high-energy trauma [4].

The optimal management of scapular fractures remains controversial, particularly regarding surgical indications [5]. Commonly proposed radiographic criteria include lateral border offset (LBO) > 20–25 mm, angular deformity > 45°, combined deformity with LBO > 15 mm and angular deformity > 30°, intra-articular step-off > 3–4 mm, glenopolar angle < 20–22°, and displaced double disruption of the superior suspensory shoulder complex (SSSC) [5,6]. However, these thresholds remain controversial and are not uniformly applied in clinical practice, particularly in fractures with combined medialization and angular deformity. In our interpretation of the existing literature, the rationale for lowering the threshold values when multiple deformity parameters coexist remains insufficiently established. Therefore, surgical indications in the present study were determined primarily according to individual deformity parameters rather than combined threshold criteria. Specifically, LBO > 20 mm was selected as the threshold for medialization deformity, whereas angular deformity > 30° was used as a relatively inclusive criterion because combined deformity criteria were not separately applied.

Among these parameters, LBO, angular deformity, and glenopolar angle are primarily used in scapular body fractures, whereas intra-articular step-off is used to evaluate glenoid fossa fractures. SSSC injuries may represent a distinct injury pattern involving both osseous and ligamentous structures and may therefore be considered separately [7,8,9,10,11,12].

Recently, Galich et al. reported favorable functional outcomes after nonoperative treatment even in extra-articular scapular fractures meeting the commonly cited Cole surgical criteria, further emphasizing the ongoing controversy regarding optimal treatment selection [13]. However, the absence of a direct comparison between operative and non-operative groups in that study limits the ability to draw definitive conclusions about the relative effectiveness of these approaches. In addition, the authors also suggested that substantial angular deformity (>40°) and lateral border displacement (>20 mm) may still negatively influence functional outcomes. These findings suggest that although the indications for surgery remain controversial, scapular malalignment itself may still have biomechanical and potential clinical importance.

Previous studies have reported favorable outcomes following operative treatment of displaced scapular fractures [14,15,16,17,18]. However, restoration of scapular alignment after surgical treatment has been less frequently evaluated quantitatively. In particular, correction of LBO and angular deformity may influence glenoid position and overall scapular alignment.

The purpose of this study was to evaluate radiologic restoration and clinical outcomes following posterior open reduction and internal fixation (ORIF) of displaced scapular fractures using computed tomography (CT)-based measurements. In addition, factors potentially associated with postoperative clinical outcomes were analyzed.

## 2. Materials and Methods

### 2.1. Study Design and Patient Selection

This retrospective case series included patients who underwent surgical treatment for scapular fractures at a single institution between 2017 and 2024. The inclusion criteria were: (1) scapular body or glenoid fractures requiring a posterior surgical approach, (2) fractures meeting radiographic criteria for operative treatment, and (3) a minimum follow-up period of at least 12 months.

Surgical treatment was indicated when one or more of the following criteria were met, based on previously reported operative indications with slight modification: (1) lateral border offset (LBO) greater than 20 mm, (2) angular deformity greater than 30°, or (3) displaced intra-articular fracture with a step-off greater than 3 mm [5,6]. A total of 20 patients met these criteria and were included in the study.

Surgical fixation was generally performed within 2 weeks after injury whenever possible. In one patient, surgery was delayed until 19 days after injury because of the need for treatment and neurologic stabilization of an associated traumatic brain injury. The mean interval from injury to surgery was 8.4 days (range, 1–19 days).

**Figure 1:**
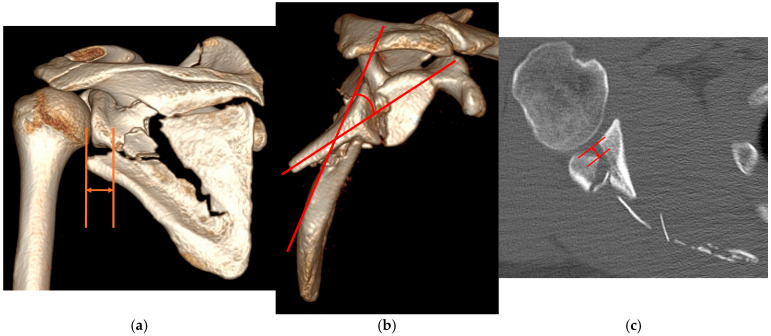
(**a**) Lateral border offset (LBO) is defined as the mediolateral displacement between the proximal and distal fragments along the lateral border of the scapula. (**b**) Angular deformity is measured as the angle between the long axis of the glenoid and the lateral border of the distal fragment. (**c**) Intra-articular step-off is defined as the maximal displacement of the glenoid articular surface.

Preoperative three-dimensional shoulder CT was used to evaluate fracture morphology and determine whether the fractures met the operative indications. Angular deformity was measured as the angle between the long axis of the glenoid and the lateral border of the distal fragment. (Figure 1).

### 2.2. Surgical Technique

All procedures were performed with the patient in the lateral decubitus position using a posterior approach. During the early period of the study, fractures requiring both lateral and medial plating were treated using the modified Judet approach [5,19], whereas fractures stabilized with lateral plating alone were managed using a direct lateral column approach (Brodsky approach). In the later period of the study, even cases requiring dual plating were treated using the Brodsky approach combined with an additional medial border approach.

**Figure 2:**
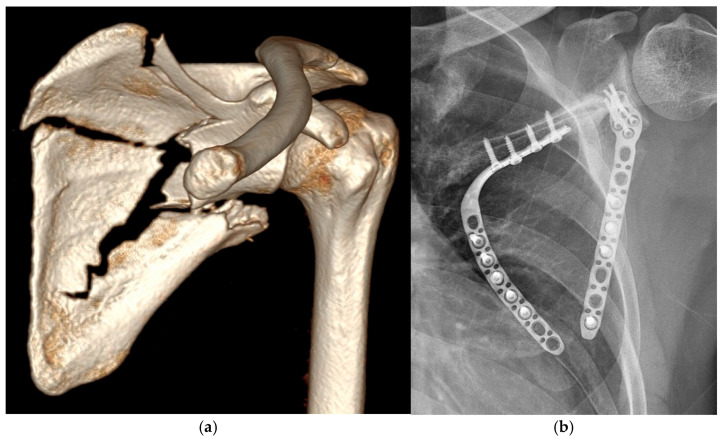
(**a**) Preoperative three-dimensional computed tomography demonstrates a large free fragment at the superomedial border of the scapula. (**b**) Postoperative radiograph shows dual plating, with a plate along the lateral border and an additional plate applied to stabilize the superomedial fragment.

In the Brodsky approach, a longitudinal skin incision was made along the lateral border of the scapula. The interval between the infraspinatus and teres minor muscles was developed to expose the glenoid and the lateral border of the scapula. Subperiosteal detachment of the infraspinatus was limited to the area required for plate placement, primarily along the posterior glenoid and lateral border. In cases where sufficient bony exposure for proximal or distal screw fixation was difficult, partial release of the medial portion of the infraspinatus and teres minor was additionally performed to improve exposure. Because the release area was generally limited, formal muscle repair was not routinely performed. During this approach, the circumflex scapular artery was ligated [20]. Additional medial plating was performed when a large superomedial free fragment could not be adequately stabilized by lateral plating alone. (Figure 2) In these cases, a curved incision was made along the scapular spine and medial border to expose the fracture site. In the modified Judet approach, after elevation through the subcutaneous layer, muscular dissection was performed similarly to the Brodsky approach and was generally limited to the lateral scapular surface. In cases requiring medial plating, additional subperiosteal detachment was limited to the medial border where the plate was positioned, and the muscle and fascia were repaired to the medial scapular border after fixation.

Implants used in the early period of the study included 3.5 mm reconstruction locking compression plates (LCPs) and 2.3–2.7 mm LCPs. In the later period, anatomically contoured scapular plates were used.

Postoperatively, patients wore an arm sling for 4 weeks. Painless arc motion was permitted beginning 2 weeks after surgery. After sling removal, passive and active ROM exercises were initiated, and muscle strengthening exercises were gradually introduced over 2–3 months postoperatively.

### 2.3. Outcome Evaluation

Radiologic evaluation of fracture reduction was performed using immediate postoperative CT scans. The degree of reduction was assessed by measuring lateral border offset, angular deformity, and intra-articular step-off. During follow-up, fracture healing and maintenance of reduction were assessed using plain radiographs, including scapular anteroposterior, outlet, shoulder Grashey, and axillary views.

Clinical outcomes were evaluated at 1 year after surgery. Shoulder range of motion (ROM) was measured, and functional outcomes were assessed using the Disabilities of the Arm, Shoulder and Hand (DASH) score [21], and the modified American Shoulder and Elbow Surgeons (ASES) score [22].

### 2.4. Statistical Analysis

All radiologic parameters were measured independently by two observers (a senior orthopedic surgeon and an orthopedic trauma fellow), and the mean values were used for analysis. Interobserver reliability was assessed using the intraclass correlation coefficient.

Preoperative and postoperative radiologic parameters were compared using the Wilcoxon signed-rank test. Clinical outcomes were compared between scapular body fractures without glenoid involvement (SBNG) and scapular body fractures with glenoid involvement (SBG) using the Mann–Whitney U test. The association between the presence of ipsilateral upper extremity injuries and clinical outcomes was also evaluated using the Mann–Whitney U test. In addition, correlations between injury severity score (ISS) [23], and clinical outcome scores were analyzed using Spearman correlation analysis. Statistical analyses were performed using IBM SPSS Statistics version 20.0 (IBM Corp., Armonk, NY, USA), and a *p*-value less than 0.05 was considered statistically significant.

## 3. Results

### 3.1. Patient Demographics and Injury Characteristics

A total of 20 patients were included in the study, consisting of 19 men and 1 woman. The mean age was 46.0 ± 12.2 years (range, 22–69 years), and the mean follow-up period was 17.3 ± 8.6 months (range, 12–40 months).

Among the fractures, 11 were classified as SBNG, whereas 9 were SBG. In all SBG cases, the fracture line extended from the glenoid into the scapular body. According to the AO/OTA classification system, these fractures were categorized as glenoid fossa fractures (type 14F) despite the presence of associated body fractures [24].

Scapular fractures were frequently associated with polytrauma. The mean injury severity score (ISS) was 25.7 ± 9.6 (range, 13–41). Ipsilateral rib fractures were present in 19 patients, and three of these patients underwent rib fixation. Associated ipsilateral upper extremity injuries included fractures of the clavicle, humerus, ulna, radius, and hand. Injuries involving the superior suspensory shoulder complex (SSSC) included process fractures, acromioclavicular joint dislocation, and distal clavicle fractures. Two patients had associated brachial plexus injuries, resulting in severe functional impairment of the affected upper extremity. One case of postoperative implant failure occurred in a patient treated with lateral plating alone using a 7-hole 2.7 mm variable-angle locking compression plate (VA-LCP). Despite this complication, all scapular fractures achieved union at final follow-up. Detailed demographic characteristics and associated injuries are summarized in Table 1.

### 3.2. Radiologic and Clinical Outcomes

**Figure 3:**
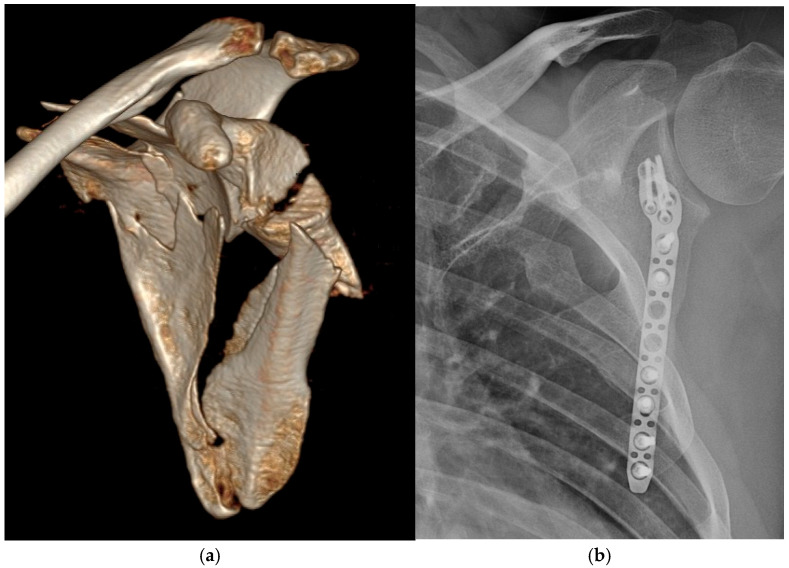
(**a**) Preoperative three-dimensional computed tomography demonstrates a fracture extending from the glenoid fossa to the scapular body. (**b**) Postoperative radiograph shows stable fixation with a lateral border plate and screw fixation of the glenoid fragment.

The mean preoperative LBO was 18.9 ± 10.7 mm (range, 0–45.5 mm), which improved to 3.1 ± 7.1 mm (range, 0–29.9 mm) postoperatively. The mean preoperative angular deformity was 28.7° ± 11.3° (range, 10.2–48.1°), which improved to 0.9° ± 3.9° (range, 0–17.8°) after surgery. The mean preoperative intra-articular step-off in the nine patients with glenoid involvement (SBG group) was 6.4 ± 2.0 mm (range, 3.7–9.7 mm), which improved to 1.8 ± 0.5 mm (range, 1–2.5 mm) postoperatively. (Figure 3) Interobserver reliability for radiologic measurements was good. The intraclass correlation coefficients were 0.80, 0.82 and 0.78 for preoperative LBO, angular deformity, and intra-articular step-off, respectively, and 0.79, 0.81, and 0.83 for postoperative LBO, angular deformity, and intra-articular step-off. Radiologic outcomes are summarized in Table 2.

At 1 year after surgery, the mean shoulder range of motion was 126° ± 34.4° (range, 10–170°) in forward flexion and 62.3° ± 20.8° (range, 0–90°) in external rotation. The mean DASH score was 27.3 ± 17.3 (range, 8.3–74.2), and the mean modified ASES score was 71.6 ± 15.0 (range, 25.8–89). After exclusion of the two patients with associated brachial plexus injury, mean forward flexion improved to 133.9° ± 22.3°, external rotation improved to 66.9° ± 15.1°, mean DASH score improved to 22.8 ± 11.5, and mean modified ASES score improved to 76.0 ± 7.5. Clinical outcomes are summarized in Table 3.

### 3.3. Subgroup and Correlation Analyses

Wilcoxon signed-rank testing demonstrated significant postoperative improvement in all radiologic parameters, including LBO (*p* = 0.001), angular deformity (*p* < 0.001), and intra-articular step-off (*p* = 0.012). Comparison of clinical outcomes between the SBNG and SBG groups using the Mann–Whitney U test showed no significant differences (DASH, *p* = 0.656; modified ASES, *p* = 0.295; VAS, *p* = 0.456). The presence of ipsilateral upper extremity injury showed a tendency toward worse functional outcomes, particularly in the modified ASES score, although this did not reach statistical significance (DASH, *p* = 0.131; modified ASES, *p* = 0.067; VAS, *p* = 0.080). After exclusion of the two patients with brachial plexus injury, the tendency toward inferior functional outcomes in patients with associated ipsilateral upper extremity injuries persisted but became less pronounced (DASH, *p* = 0.247; modified ASES, *p* = 0.126; VAS, *p* = 0.093).

Conventional plates were used in six patients, whereas anatomically contoured plates were used in fourteen patients. No statistically significant differences in DASH score (*p* = 0.433), modified ASES score (*p* = 0.482), or VAS score (*p* = 0.400) were observed between these groups.

Patients were additionally subdivided into modified Judet (*n* = 2), Brodsky-only (*n* = 16), and combined Brodsky with medial plating (*n* = 2) groups. No statistically significant differences were observed among these groups in DASH score (*p* = 0.383), modified ASES score (*p* = 0.264), or VAS score (*p* = 0.135).

Sixteen patients underwent lateral plating alone, whereas four patients underwent combined lateral and medial plating because of a large superomedial free fragment. Preoperative angular deformity tended to be greater in the combined plating group (35.4° ± 13.2°, range 17.5–48.1°) than in the lateral plating alone group (27.1° ± 10.9°, range 10.2–45.2°), although the difference was not statistically significant (*p* = 0.186). Preoperative LBO was not significantly different between the two groups (19.5 ± 13.2 mm, range 0–38.5 mm vs. 16.4 ± 21.6 mm, range 0–45.5 mm; *p* = 0.598). No statistically significant differences in DASH score (*p* = 0.345), modified ASES score (*p* = 0.394), or VAS score (*p* = 0.218) were observed between the two fixation groups.

Spearman correlation analysis demonstrated no significant association between ISS and clinical outcomes (DASH: rho = 0.133, *p* = 0.577; modified ASES: rho = −0.081, *p* = 0.736; VAS: rho = 0.160, *p* = 0.501).

## 4. Discussion

The principal finding of this study was that posterior open reduction and internal fixation (ORIF) of scapular fractures resulted in significant restoration of scapular alignment and articular reduction, with satisfactory clinical outcomes at 1 year. In particular, LBO, angular deformity, and intra-articular step-off were significantly improved after surgery. These findings suggest that posterior fixation is effective for restoration of scapular alignment in displaced scapular fractures.

Although favorable outcomes after operative treatment of scapular fractures have been increasingly reported [14,15,16,17,18], consensus regarding surgical indications remains limited. Quantifying the degree of deformity that justifies surgical intervention remains challenging. In the present study, we focused particularly on restoration of LBO and correction of angular deformity as important surgical objectives in scapular body fractures, while articular reduction was considered the primary goal in glenoid fractures.

Restoration of LBO may be clinically relevant because medialization of the glenoid can alter shoulder biomechanics, potentially affecting rotator cuff tension and posterior deltoid function [25,26]. These concepts have been indirectly discussed in the reverse shoulder arthroplasty literature [27,28,29,30]. However, because the rotator cuff is generally preserved in scapular fractures, this comparison should be interpreted cautiously and regarded only as a conceptual analogy.

Correction of angular deformity may also be clinically important because it may restore the orientation of the rotator cuff muscles. In scapular body fractures involving the glenoid, the proximal fragment frequently tilts anteriorly, resulting in rotation of the glenoid within the scapular plane. This deformity may alter the functional vectors of the supraspinatus, infraspinatus, and teres minor muscles, potentially influencing their contribution to shoulder motion. Although these biomechanical implications remain theoretical, they provide a possible rationale for correcting angular deformity during surgical treatment.

Previous studies have emphasized glenopolar angle (GPA) as an important radiographic parameter in scapular fractures [6,31,32,33]. However, in our series, only one patient had a GPA below the commonly suggested surgical threshold. In addition, GPA measurements obtained from three-dimensional CT reconstruction may still be influenced by scapular orientation and reconstruction alignment, which may reduce measurement reliability. Substantial deformities in LBO and angular deformity were more frequently observed in the present cohort, and restoration of scapular alignment through correction of these parameters did not necessarily produce large changes in GPA. Structurally, a decrease in GPA often reflects adduction deformity and medialization of the glenoid fragment. However, because the long head of the triceps brachii inserts at the infraglenoid tubercle, traction forces acting on the glenoid fragment may preferentially produce abduction rather than adduction deformity in scapular body fractures. This proposed biomechanical mechanism remains speculative and should be interpreted as a hypothesis rather than an established explanation. This biomechanical hypothesis has not been validated in cadaveric or computational studies and requires further investigation. This biomechanical tendency may partly explain why GPA did not significantly decrease despite substantial displacement and deformity in other radiologic parameters. Therefore, we considered GPA as a secondary indicator of glenoid medialization rather than an independent surgical indication.

The present cohort included both intra-articular glenoid fractures and extra-articular scapular body fractures, which may represent heterogeneous injury patterns with partially different surgical objectives. However, isolated intra-articular glenoid fractures without associated scapular body involvement were uncommon, and combined intra-articular and scapular body fractures were frequently observed in the SBG group. In addition, posterior fixation with lateral border stabilization and postoperative CT-based evaluation of scapular alignment restoration were commonly applied in both groups. Therefore, the fractures were analyzed within a unified radiologic framework centered on restoration of scapular anatomy and alignment. Nevertheless, the relatively small subgroup sample sizes limited statistical power and precluded definitive conclusions regarding differences between fracture types.

Associated injuries also appeared to influence postoperative functional outcomes. Two patients with associated brachial plexus injury demonstrated markedly inferior postoperative function, suggesting that neurologic injury may substantially affect clinical outcomes regardless of fracture reduction. These two patients demonstrated the lowest postoperative ROM values in the cohort, including forward flexion of 10° and external rotation of 0°, thereby substantially influencing the overall range of postoperative ROM measurements. Sensitivity analysis excluding these two patients demonstrated improved postoperative ROM and functional outcome scores, further suggesting that brachial plexus injury substantially influenced the overall clinical results independently of scapular reduction quality. Most associated upper extremity fractures recovered with time, whereas neurologic deficits persisted and continued to impair shoulder function at final follow-up. Both patients with brachial plexus injury were managed conservatively with observation rather than surgical intervention for the neurologic injury. Previous studies have generally reported more favorable postoperative functional outcomes after operative treatment of scapular fractures [16,17,18]. In comparison, the present cohort demonstrated relatively inferior DASH and modified ASES scores. These factors may partly explain why the postoperative DASH and modified ASES scores in the present study were inferior to those reported in several previous studies of operatively treated scapular fractures. In addition, the relatively shorter follow-up duration in the present cohort compared with previous studies may also have contributed, because postoperative functional recovery may continue to improve over time after scapular fracture surge ry. Furthermore, the inclusion of the implant failure case in the overall cohort likely additionally influenced the postoperative functional outcome scores. Because DASH reflects global upper-extremity disability rather than isolated shoulder function, interpretation of DASH scores in this cohort should be performed cautiously, particularly in patients with associated neurologic or major ipsilateral upper-extremity injuries. In contrast, the modified ASES score may better reflect shoulder-specific functional outcomes related to scapular fracture reduction and restoration of scapular alignment.

One case of implant failure occurred in a severely displaced fracture treated with lateral plating alone using a relatively small 7-hole 2.7 mm variable-angle locking compression plate (VA-LCP). Although fracture union was eventually achieved without revision surgery, residual deformity and unsatisfactory clinical outcome remained. This patient demonstrated the largest residual postoperative LBO (29.9 mm) in the cohort, representing a radiographic outlier that substantially influenced the postoperative range of alignment measurements. This case highlights the importance of maintaining postoperative scapular alignment through adequate fixation strength in highly displaced fractures. In retrospect, stronger fixation strategies, such as a 3.5 mm plate or dual plating, may have been more appropriate for this fracture pattern.

This study has several limitations. First, the retrospective design introduces potential sources of bias. Second, the sample size was relatively small, and the cohort was heterogeneous, including both intra- and extra-articular fractures as well as associated injuries, which may have influenced the clinical outcome analyses. In particular, several subgroup analyses demonstrated clinically meaningful trends without reaching statistical significance, suggesting the possibility of type II error due to limited statistical power. In addition, because multiple subgroup comparisons were performed without formal correction for multiple testing, the possibility of inflated type I error should also be considered. Therefore, these subgroup analyses should be interpreted as exploratory and hypothesis-generating, and the reported *p*-values should be interpreted with substantial caution, particularly given the relatively small subgroup sizes. Third, because standardized contralateral shoulder ROM and functional outcome data were not consistently available, direct comparison with the uninjured side could not be performed. In addition, the absence of a nonoperative control group limited the ability to directly attribute postoperative clinical outcomes specifically to surgical intervention. Therefore, the present study should primarily be interpreted as an analysis of radiologic restoration following posterior fixation rather than definitive evidence of functional superiority over nonoperative treatment. Furthermore, the relatively limited follow-up duration may also have reduced the ability to detect potential long-term functional differences between fracture subgroups, including glenoid involvement. Fourth, fixation constructs, implants, and surgical approaches evolved during the study period. Combined lateral and medial plating was generally used in fractures with greater angular deformity and large superomedial free fragments, introducing potential confounding by indication. Although exploratory subgroup analysis demonstrated no statistically significant differences in DASH, modified ASES, or VAS scores according to fixation construct, these analyses were substantially underpowered because of the relatively small subgroup size. Fifth, postoperative MRI or delayed CT evaluation of rotator cuff muscle atrophy or fatty infiltration was not routinely performed. Therefore, the potential effects of surgical muscle detachment and release on postoperative muscle quality and external rotation function could not be adequately evaluated. Finally, the biomechanical implications of restoration of scapular alignment remain indirect and require further biomechanical and clinical investigation.

## 5. Conclusions

Posterior ORIF restored scapular alignment and articular congruity in displaced scapular fractures. Significant corrections of LBO, angular deformity, and intra-articular step-off were achieved following surgery. Restoration of scapular alignment may represent an important consideration in the surgical treatment of displaced scapular fractures. However, because of the retrospective design, relatively small and heterogeneous cohort, and absence of a nonoperative comparison group, the present findings should be interpreted cautiously and regarded as hypothesis-generating. Further larger, ideally multicenter, prospective comparative studies are required to clarify the clinical significance of restoration of scapular alignment.

## Figures and Tables

**Table 1:** Patient demographics, injury characteristics, and fracture classification.

No .	Sex	Age	Injury Mechanism	* ISS	Classifications of Scapular Fracture		Ipsilateral Rib Fractures	Ipsilateral Upper Extremity Injury
					AO/OTA	Ada and Miller		
1	M	22	Motorcycle collision	18	14B(x)2(ls)	IIA	Yes	
2	M	46	Pedestrian struck by a car	13	14F0[B1(l)]	IIA	No	
3	M	40	Driver in a motor vehicle collision	29	14B2(lm)	IIC	Yes	
4	F	69	Pedestrian struck by a car	24	14F1.3(p)[B1(l)]	III	Yes	
5	M	47	Driver in a motor vehicle collision	36	14F2.1[B2(lms)]	III	Yes	
6	M	32	Motorcycle collision	38	14B(y)2(lms)	IIA	Yes	
7	M	25	Motorcycle collision	14	14F1.3(e)[B2(lms)]	III	Yes	
8	M	49	Pedestrian struck by a car	38	14B1(lm)	IIC	Yes	Humerus shaft fracture Ulna shaft fracture
9	M	35	Motorcycle collision	21	14F1.3(e)[B2(lms)]	III	Yes	
10	M	55	Caught in machinery	17	14F(x)1.3(e)[B1(l)]	III	Yes	Distal clavicle fracture Brachial plexus injury
11	M	33	Struck by a falling machine	41	14B(y)2(lms)	IIB	Yes	
12	M	58	Fall from height	13	14F1.3(i)[B1(ms)]	III	Yes	Clavicle shaft fracture
13	M	53	Motorcycle collision	22	14B2(lms)	IIB, IIC	Yes	Radius shaft fracture
14	M	60	Struck by an excavator bucket	17	14B2(lm)	IIC	Yes	Clavicle shaft fracture Brachial plexus injury
15	M	60	Motorcycle collision	38	14F(x)1.3(e)[B2(ms)]	III	Yes	Acromioclavicular joint injury Ulnar shaft fracture 5th metacarpal neck fracture
16	M	46	Crushed by a truck	29	14B2(lms)	IIB, IIC	Yes	
17	M	41	Motorcycle collision	14	14B1(lm)	IIC	Yes	Clavicle shaft fracture
18	M	40	Motorcycle collision	36	14B1(lm)	IIC	Yes	Acromioclavicular joint injury Distal radioulnar fracture 1st distal phalanx fracture
19	M	50	Struck by a falling glass panel	34	14B2(lms)	IIB, IIC	Yes	Clavicle shaft fracture Thumb amputation
20	M	59	Fall from height	22	14F1.3(i)[B2(lms)]	III	Yes	

* Injury Severity Score.

**Table 2:** Preoperative and postoperative radiologic parameters.

	* Preoperative	* Postoperative	^†^ *p* Value
Lateral border offset (mm)	18.9 ± 10.7 (0–45.5)	3.1 ± 7.1 (0–29.9)	0.001
Angular deformity (°)	28.7 ± 11.3 (10.2–48.1)	0.9 ± 3.9 (0–17.8)	<0.001
^‡^ Intra-articular step-off (mm)	6.4 ± 2.0 (3.7–9.7)	1.8 ± 0.5 (1–2.5)	0.012
Glenopolar angle (°)	39.4 ± 12.0 (20.8–63.4)	36.4 ± 8.8 (18.7–49.6)	0.070

* Values are presented as means ± standard deviations (range). ^†^ *p* values were calculated using the Wilcoxon signed-rank test. ^‡^ Intra-articular step-off measurements were analyzed only in nine patients with glenoid involvement.

**Table 3:** Clinical outcomes and subgroup analysis.

		Number of Patients	Mean ± Standard Deviations (Range)	^‖^ *p* Value
* DASH score	All	20	27.3 ± 17.3 (8.3–74.2)	
	^†^ SBNG	11	27.6 ± 15.5 (9.2–61.7)	0.656
	^‡^ SBG	9	26.9 ± 21.2 (8.3–74.2)	
	With ^§^ UEI	9	35.7 ± 20.4 (9.2–74.2)	0.131
	Without UEI	11	20.4 ± 10.8 (8.3–39.2)	
Modified * ASES score	All	20	71.6 ± 15.0 (25.8–89)	
	SBNG	11	69.6 ± 16.4 (25.8–87.5)	0.295
	SBG	9	74.0 ± 14.7 (38.3–89.0)	
	With UEI	9	64.3 ± 19.6 (25.8–87.5)	0.067
	Without UEI	11	77.5 ± 7.1 (66.0–89.0)	
* VAS	All	20	3.1 ± 1.4 (1–8)	
	SBNG	11	3.4 ± 1.6 (2.0–8.0)	0.456
	SBG	9	2.8 ± 1.1 (1.0–5.0)	
	With UEI	9	3.8 ± 1.8 (2.0–8.0)	0.080
	Without UEI	11	2.5 ± 0.7 (1.0–3.0)	
Range of motion	Forward flexion		126 ± 34 (10–170)	
	Extension		32.3 ± 14.8 (0–55)	
	Abduction		120 ± 24.3 (60–150)	
	Adduction		27.5 ± 7.0 (0–30)	
	Internal rotation		29.8 ± 8.2 (15–40)	
	External rotation		62.3 ± 20.8 (0–90)	

* Disabilities of the Arm, Shoulder and Hand, American Shoulder and Elbow Surgeons, Visual analog scale. ^†^ Scapular body fracture without glenoid involvement. ^‡^ Scapular body fracture with glenoid involvement. ^§^ Upper extremity injury. ^‖^ *p* values were calculated using the Mann–Whitney U test.

## Data Availability

The datasets used and/or analyzed during the current study are available from the corresponding author on reasonable request.

## Abbreviations

The following abbreviations are used in this manuscript:

LBO	Lateral Border Offset
ROM	Range Of Motion
DASH	Disabilities of the Arm, Shoulder and Hand
ASES	American Shoulder and Elbow Surgeons
SSSC	Superior Suspensory Shoulder Complex
CT	Computed Tomography
LCP	Locking Compression Plate
SBNG	Scapular Body fracture without Glenoid involvement
SBG	Scapular Body fracture with Glenoid involvement
ISS	Injury Severity Score
GPA	Glenopolar Angle
ORIF	Open Reduction and Internal Fixation
